# Overcoming the Underutilisation of Peritoneal Dialysis

**DOI:** 10.1155/2015/431092

**Published:** 2015-11-11

**Authors:** Jernej Pajek

**Affiliations:** Department of Nephrology, University Medical Centre Ljubljana, Zaloška 2, SI-1525 Ljubljana, Slovenia

## Abstract

Peritoneal dialysis is troubled with declining utilisation as a form of renal replacement therapy in developed countries. We review key aspects of therapy evidenced to have a potential to increase its utilisation. The best evidence to repopulate PD programmes is provided for the positive impact of timely referral and systematic and motivational predialysis education: average odds ratio for instituting peritoneal dialysis versus haemodialysis was 2.6 across several retrospective studies on the impact of predialysis education. Utilisation of PD for unplanned acute dialysis starts facilitated by implantation of peritoneal catheters by interventional nephrologists may diminish the vast predominance of haemodialysis done by central venous catheters for unplanned dialysis start. Assisted peritoneal dialysis can improve accessibility of home based dialysis to elderly, frail, and dependant patients, whose quality of life on replacement therapy may benefit most from dialysis performed at home. Peritoneal dialysis providers should perform close monitoring, preventing measures, and timely prophylactic therapy in patients judged to be prone to EPS development. Each peritoneal dialysis programme should regularly monitor, report, and act on key quality indicators to manifest its ability of constant quality improvement and elevate the confidence of interested patients and financing bodies in the programme.

## 1. Introduction

**Figure 1:**
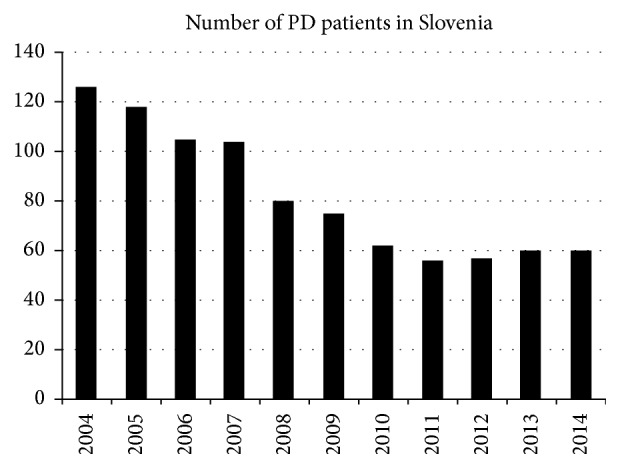
Dropping number of PD patients in the example of Slovenia's national PD cohort for the time period from 2004 to 2014.

Over the last 15 years the proportion of all dialysis patients treated with peritoneal dialysis (PD) declined significantly in developed countries [1]. Slovenia is a good example of such a negative trend with the numbers of PD patients dropping significantly in the period from 2004 to 2014 (Figure 1).

A general shift towards a higher age at start of dialysis treatment and increasing comorbidity cannot in whole explain the causality of this problem [2]. Other possible factors affecting declining PD utilisation are proliferation of haemodialysis (HD) units and private dialysis provider penetration in some healthcare systems, both factors being associated with lower use of PD [3, 4]. Further impact on PD penetration may have come from insufficient patient education and physician bias [5]. A special concern with reducing numbers of patients is a possible (and probable) decrement in experience, expertise, and quality of PD programmes including the loss in quantity and quality of training for medical staff (physicians and nurses). Data from the USA raised concerns that there are an insufficient number of PD patients and allocation of time available for trainees in PD [6]. This may cause a further decline in PD utilisation thus starting a negative spiral for this dialysis modality.

The fall in PD utilisation is a concern since PD is a precious renal replacement modality that offers patients the convenience of home treatment, flexible schedule and increased freedom perception, less haemodynamic instability issues, and higher quality of life [7]. PD abolishes the inconvenience and costs of patient transport associated with in-centre haemodialysis. Further benefits of PD are associated with residual renal function preservation [8], lower hospitalisation and access intervention rates [9, 10], and perhaps better short-term outcome after transplantation [11, 12]. PD is able to provide equal outcomes as haemodialysis [13] and it may save lives when vascular access is exhausted. It is clear that the fall in utilisation of PD should be prevented; however there is no clear consensus on the actions that have to be taken and the responsibility of the governing bodies for implementation of these actions. Here we present several key opportunities and strategies for revitalisation of PD programmes with a special emphasis on their feasibility and published evidence.

## 2. Timely Referral and Predialysis Education

In Europe there is a domination of haemodialysis (HD) as a starting modality in late-referred chronic kidney disease (CKD) patients [14]. Late referral is associated with several well-known detrimental factors in advanced CKD: lost opportunity to slow CKD progression and to properly relieve CKD complications [15–17], lower rate of transplantation [18], deprivation of a proper choice of dialysis modality, and higher mortality [14, 19]. Prevention of late referral should include actions on a patient social level (improvement in education and income level, proper health insurance) and health-system levels (improvement of communication between referring physicians and nephrologists, education of referring physicians about the appropriate timing of referral), since these two categories of factors are both associated with late referral [20]. Since predialysis education may be associated with improved survival [21, 22], one of the most important additional benefits of timely referral is the opportunity for execution of a proper predialysis education.

A significant impact of predialysis education or timely referral on the choice of peritoneal dialysis is shown in the summary in Table 1. Motivating patients to start with peritoneal dialysis takes time and persuasive talent from the dialysis team and confidence and comprehension from the patient, which are all often absent in the late-referred patients [23]. As shown in Table 1 the impact of intensive or at least timely education and information on modality choice has so far only been demonstrated in retrospective studies. However with all the information about the benefits of timely predialysis referral, counselling, and education, undertaking the prospective randomised trial would seem unnecessary and unethical. The observational studies have been consistently showing that with predialysis education the proportion of patients choosing peritoneal dialysis increased and reached relatively high levels. An overview of data in Table 1 shows that the average odds ratio of choosing PD versus HD with timely predialysis education is 2.6 across the cited studies.

## 3. Unplanned Acute Start of Peritoneal Dialysis

Unplanned and suboptimal initiation is the term proposed to include dialysis initiation in hospital and/or with a central venous catheter (CVC) and/or with a patient not starting on their chronic modality of choice [24]. Rates of unplanned starts of dialysis are reported to be in the range of 24–49% in the survey of eight European studies [24]. Except for the units with established teams with skills for acute unplanned start of PD, the vast majority of unplanned cases are managed by placing a CVC and the first dialysis setting that these patients experience is a HD unit. It is a commonly held perception that once started on HD, the patients have a tendency to continue with this modality and there are a significantly lower number of patients treated with PD than HD even after clinical stabilisation in unplanned dialysis starters [25]. Although at least part of excess mortality risk for HD patients dialysed through CVCs may be attributable to inferior catheter based vascular access [26], substantial number of patients may rely on this vascular access even after several months as the median time to fistula use from dialysis start may be more than 4 months [27]. On the other hand, units with established acute-start PD programmes can offer patients an alternative way to start dialysis treatment; however such a programme needs careful planning, dedication, and skills to be successful.

Acute unplanned start of peritoneal dialysis is generally offered to patients in two clinical scenarios: the patient had previously been given some information on dialysis modality and he opted for PD before the unexpected fast deterioration in kidney function happened or after a brief discussion in the hospital about the renal replacement modalities the patient finds peritoneal dialysis acceptable. Provided there is no uremic encephalopathy, pericarditis or colitis, severe hyperkalemia or pulmonary congestion, or another factor demanding dialysis sooner than within 48 hours, acute unplanned start of peritoneal dialysis is a feasible, effective, and safe option.

The PD catheter should be placed as soon as possible and an early start of PD with low fill volumes (750–1000 mL), automated PD tidal regime with a cycler in supine position can be started. The treatment time is variable, from 6 to 12 hours [28]. With such a start the proportion of early leaks along the catheter was reported to be 7.7% (4 out of 52 patients) and the total incidence of catheter dysfunction was 15.4% as compared to 5.8% in the control group with PD start at least 12 days after PD catheter placement [29]. The current practice of delaying the PD start for at least 2 weeks after catheter implantation (but for most patients clinicians may try to wait for 4–6 weeks) is based on a low level of evidence and currently there is a randomised research study in flow comparing the early start of PD 7 days after catheter insertion to later time points of PD start [30]. Early start of peritoneal dialysis enables increased utilisation of peritoneal dialysis in suboptimal initiation conditions and offers an escape from the complications associated with interim HD and presence of CVCs [27].

## 4. Peritoneal Catheter Insertion by Nephrologists

Dedicated catheter insertion team available 24/7 is the necessary condition for acute unplanned start of peritoneal dialysis. If there is an experienced and dedicated nephrologist performing catheter insertions available at the dialysis unit many logistic and operative schedule barriers for PD catheter insertion (such as competition for limited procedural rooms) may be more easily tackled. The set-up of interventional nephrology catheter insertion service was reported to enable growth of PD programmes [31]. The inclusion of interventional nephrologist catheter placement in the integrated care approach to dialysis start has resulted in a relatively large PD penetration of 44.8% in one of the reports [32]. Another study reported an increase in the prevalence of PD from the relative share of 16–18% to 22–32% [33]. Catheter implantation by nephrologists compared to surgical or radiological services was associated with higher rates of successfully finalised peritoneal dialysis utilisation in patients undergoing elective PD catheter insertion [34]. The Brazilian experience has shown similar outcomes and success of catheter implantation by interventional nephrologists and surgeons [35]. On the other hand the opinion has been expressed that the placement of PD catheters should optimally be done by surgeons using advanced laparoscopic techniques [36] due to ability to perform rectus sheath tunnelling, omentopexy, and adhesiolysis [37] making this issue a controversial one.

At some dialysis centres (including the author's) there are a long-term experience and positive results with divided care for establishment of vascular access for HD between interventional nephrologists and vascular surgeons (the bulk of operations being performed by interventional nephrologists [38]). It may be that such a model could prove to be optimal also for peritoneal access, the interventional nephrologists taking care of first implantation in cases without expected complications or adhesions and abdominal surgeons performing the access in demanding cases necessitating laparoscopy, adhesiolysis, hernia repair, cholecystectomy, and other cases necessitating general anaesthesia. In any case, PD programme leaders should gain good support for establishment of interventional nephrology service in PD catheter placement from hospital managers, lead clinicians, surgical teams, and the practicing nephrology team. With this it will be possible to train devoted nephrologists and maintain the number of procedures necessary for maintenance of skill and service quality.

## 5. Assisted Peritoneal Dialysis for Frail and Dependant Patients

The patient population reaching end-stage CKD is growing in age, frailty, comorbidity, and dependance. This is one of the major obstacles for institution of PD as it is a form of self-delivered home based therapy. The overwhelming association of having a strong social support network and being functionally able with choosing PD emphasizes the need for assisted PD [39]. The French experience published in 2006 has shown that patients on assisted PD were on average 74 years old, 22 years older than others, and had higher comorbidity and hospitalisation rate [40]. A Canadian survey has shown that the most prevalent conditions that act as barriers to self-care PD in elderly patients are exactly the ones that can be overcome by home assistance: decreased strength to lift PD bags, decreased dexterity or vision, anxiety, decreased cognition, and immobility [41]. In this study the probability of being considered eligible for PD significantly increased in the regions with home care assistance programme available. The indications for assisted PD use may be broadened from patients with physical and cognitive disabilities to patients with exhausted vascular access and haemodynamic instability during HD, thus likely extending the lives of those patients [42]. The possibility of assisted PD and family support was shown to increase PD utilisation from 23 to 39% among patients with barriers to self-care in a Canadian centre [43]. Technique failure and peritonitis rates were in general within acceptable limits and independent of the method of assistance (done by either nurses or family members) [44, 45]. The possibility of having periods without assistance (e.g., the family provides assistance on weekends or helps with disconnections) enables assisted PD to become more cost-effective although elevated costs of reimbursed nursing assistance are a serious concern [46]. Training of staff at nursing homes for PD delivery is an additional area of a possible increment in utilisation of PD.

## 6. Encapsulating Peritoneal Sclerosis Prevention

“There is no evidence to withhold PD as a treatment option because of fear of development of EPS” was the final conclusion of an ISPD statement on length of time on PD and encapsulating peritoneal sclerosis (EPS) [47]. Although the major opinion has diverged from the proposal that simple peritoneal sclerosis is just a stage towards the development of EPS and if left enough time, all patients would sooner or later develop EPS [48], there is still doubt and anecdotal communication between nephrologists still reflects the fear of EPS as one of the major unavoidable detrimental factors when considering starting or maintaining patients on PD. The concept of “expiry date” for PD after 5 or so years still seems viable among nephrologists. So the crucial question to overcome this fear is this: what can we offer our patients on PD to prevent EPS?

The usage of new biocompatible solutions is associated with stabilisation of peritoneal transport rate [49], lower peritonitis rates [50], and improved histology with less fibrosis and vascular sclerosis [51, 52]. These are all risk factors associated with emergence of EPS, so the usage of biocompatible solutions might be one way towards reducing the risk of this complication. Lowering the peritoneal glucose exposure is a prudent task to ensure stability of peritoneal membrane [53] and protecting residual renal function may help in accomplishing this goal. The inhibition of renin-angiotensin system is additional therapy that should probably be offered to all PD patients that tolerate this treatment, due to its protective effects on the actions of transforming growth factor-beta [54], aldosterone, and deposition of collagen [55] and plasminogen activator inhibitor-1 level [56]. Beta-blockers should perhaps be excluded from the antihypertensive therapy [57].

After 4-5 years of treatment, the patients who are identified as EPS prone (increasing speed of peritoneal transport, severe infectious peritonitis with haematoperitoneum, overexposure to glucose, or ultrafiltration failure) may be treated with prophylactic tamoxifen [48, 58] and glucocorticoids in cases of sterile inflammatory peritoneal syndrome manifestations (unspecific abdominal pain, modestly elevated inflammatory markers (i.e., CRP) without another apparent cause, and worsening of nutritional status). An additional measure in long-term PD patients at EPS risk is the possibility of combining PD and HD therapy, to lower the glucose exposure, and avoiding abrupt termination of PD, which is a known possible second hit in the two-hit hypothesis of EPS development [59]. In EPS prone patients after renal transplantation, early minimisation or discontinuation of calcineurin inhibitors, institution of mTOR inhibitors, and maintaining glucocorticoids for at least 6–12 months are suggested as the best immunosuppressive strategy [60].

To properly monitor the patients on PD regular measurement of peritoneal membrane transport status is recommended. In patients with consistent rise in the speed of small solute transport, effluent carcinomic antigen-125 (CA-125) and interleukin-6 (IL-6) can be monitored as well [61]. The combination of longer time on PD (above 4-5 years), with sustained rise in speed of transport (D/P for creatinine), effluent IL-6, and a fall in effluent CA-125 should prompt the clinician to perform imaging study (CT scan is the current imaging technique of choice) and to consider prophylactic therapy and a possible conversion to HD. Before converting to HD great emphasis must be put on establishing vascular access since in general patients converted to HD via CVCs tend to do worse than patients staying on PD [62]. In patients not in fibrotic EPS phase, the Japanese authors propose maintaining the PD catheter and performing peritoneal lavage to offer an escape from additional “hit” of catheter removal and opportunity to monitor effluent levels of fibrin, IL-6, and CA-125. This is used to judge the success of prophylactic therapy and to more easily decide on the proper timing of catheter removal, when the levels of inflammatory markers in the effluent decrease [63, 64].

## 7. Constant Quality Monitoring and Improvement

There are several key parameters which are universally accepted as quality of service indicators in the field of peritoneal dialysis. Peritonitis rates below 1 episode in 18 patient-months [65] and* Staphylococcus aureus* catheter infection rates below 1 in 240 patient-months [66] are well established minimal quality indicators. PD programmes may wish to regularly monitor and act on some additional indicators such as technique failure and its causes, peritoneal infection causative agents and their susceptibility to antibiotics, mean haemoglobin levels and epoetin usage, mean phosphate serum levels, and perhaps EPS incidence. PD programmes able to manifest their updated results and quality indicators may be more easily benchmarked and be able to express a larger self-confidence in predialysis information given to patients. This would also help to balance physician and nurse bias towards HD. A PD programme with satisfactory and constantly improving quality indicators can be more readily and boldly advertised as a viable and good option for renal replacement therapy.

## 8. Conclusion

**Figure 2:**
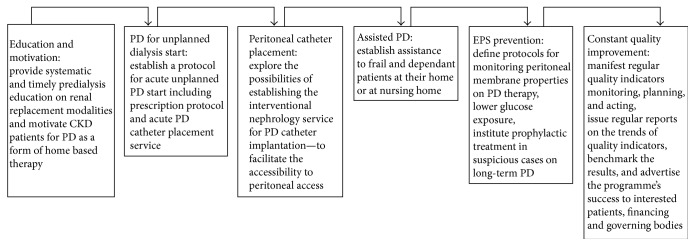
Summary of key steps in overcoming the underutilisation of peritoneal dialysis programmes.

PD should be regarded as a safe and efficient form of renal replacement modality; however the declining numbers of patients in PD programmes in developed countries are a cause for concern. Several strategies summarized in Figure 2 can be pursued to reverse this unfavourable course. Timely referral and proper predialysis education are two crucial factors with the largest potential to repopulate PD programmes. Clinicians should consider using PD not only in planned but also for unplanned-suboptimal dialysis starts. This would be possible and easier in units having interventional nephrologists providing catheter implantation service. The possibility of offering assisted peritoneal dialysis to elderly frail patients should be a part of a modern PD programme, since these are the patients whose quality of life on replacement therapy may benefit most from assisted modality performed at patient's home. The concept of “expiry date for PD” should be abandoned and replaced by the close monitoring, preventing measures, and timely prophylactic therapy in patients judged to be prone to EPS development. The PD programmes should regularly monitor, report, and act on key quality indicators which would give them a higher level of confidence towards not only interested patients seeking optimal renal replacement care, but funding and governing authorities as well.

## Figures and Tables

**Table 1:** Summary of studies on the impact of predialysis education in modality choice.

Reference	Study type (number of patients)	Number of patients with structured/timely educational intervention versus controls	Modality choice (PD versus HD)
Ahlmén et al., 1993 [67]	Retrospective single-centre cohort (*N* = 101)	N/A (all patients invited to education)	38% chose PD versus 24% choosing HD

Prichard, 1996 [68]	Retrospective single-centre cohort (*N* = 150)	N/A (all patients exposed to an extensive education programme)	Of 74 patients with a free modality choice 50% chose PD

Little et al., 2001 [69]	Retrospective single-centre cohort (*N* = 254)	65% with timely counselling versus 35% counselled at or after dialysis start	50.9% chose PD versus 34.8% of controls

Marrón et al., 2005 [70]	Retrospective multicentre observational (*N* = 626)	37% versus 63%	31% chose PD versus 8.3% of controls

Ribitsch et al., 2013 [71]	Retrospective single-centre cohort (*N* = 227)	30.8% versus 69.2%	54.3% chose PD versus 28% of controls
